# Delegation ärztlicher Leistungen an rheumatologische Fachassistenten: Effekte auf Depression und Angst bei Patienten mit rheumatoider Arthritis

**DOI:** 10.1007/s00393-023-01403-9

**Published:** 2023-08-30

**Authors:** Juliana Rachel Hoeper, Florian Schuch, Patricia Steffens-Korbanka, Georg Gauler, Martin Welcker, Jörg Wendler, Ulrich von Hinüber, Sara Eileen Meyer, Andreas Schwarting, Jan Zeidler, Torsten Witte, Dirk Meyer-Olson, Kirsten Hoeper

**Affiliations:** 1 Center for Health Economics Research Hannover (CHERH), Leibniz Universität Hannover, Hannover, Deutschland; 2 Klinik für Rheumatologie und Immunologie, Medizinische Hochschule Hannover, Carl-Neuberg-Strasse 1, 30625 Hannover, Deutschland; 3 Rheumatologie, Internistische Praxisgemeinschaft, Erlangen, Deutschland; 4 Rheumapraxis an der Hase, Osnabrück, Deutschland; 5 MVZ für Rheumatologie, Planegg, Deutschland; 6 Praxis für Rheumatologie und Osteologie, Hildesheim, Deutschland; 7 Klinik für Rheumatologie und klinische Immunologie, Universitätsmedizin Mainz, Mainz, Deutschland; 8 ACURA Kliniken Rheuma-Akutzentrum Rheinland-Pfalz, Bad Kreuznach, Deutschland; 9 Rheumatologie, m&i Fachklinik Bad Pyrmont, Bad Pyrmont, Deutschland

**Keywords:** Screening, Ambulante Versorgung, Komorbidität, Krankheitsverlauf, Teambasierte Versorgung, Screening, Outpatient care, Comorbidity, Disease process, Team based care

## Abstract

**Hintergrund:**

Bei 80 % der Patienten mit einer rheumatoiden Arthritis (RA) tritt mindestens eine Komorbidität auf. Neben kardiovaskulären Komorbiditäten sind psychische Erkrankungen häufig. Die Prävalenz von Depression und Angst ist bei Betroffenen höher als in der Allgemeinbevölkerung. Ein Screening auf Komorbidität ist hoch relevant. Die Unterversorgung im fachärztlichen Bereich lässt dies kaum zu. Die Implementierung einer Visite durch die rheumatologische Fachassistenz (RFA) bietet Potenzial, die Versorgung zu verbessern und der Unterversorgung zu begegnen.

**Fragestellung:**

Ziel ist, Auswirkungen einer teambasierten Versorgung auf den Verlauf von Depression und Angst bei Patienten mit einer seropositiven RA im Krankheitsschub zu untersuchen.

**Material und Methoden:**

Es handelt sich um eine multizentrische, pragmatische, randomisierte, kontrollierte Studie über 1 Jahr mit 224 Patienten. Nach Baseline folgen 5 Visiten. In der Interventionsgruppe (IG) fanden 3 zunächst bei der RFA statt. Depression, Angst und Behandlungszufriedenheit werden genauer betrachtet.

**Ergebnisse:**

In der IG hat sich die ängstliche Symptomatik über 12 Monate signifikant verbessert (*p* = 0,036). Auch die Anteile der Patienten mit Ängstlichkeit verändern sich signifikant in der Interventionsgruppe (*p* < 0,001), während es in der Kontrollgruppe im Vergleich zwischen Baseline und Monat 12 zu keiner Veränderung kam. Die Werte der Depressionsskala unterschieden sich nicht signifikant (*p* = 0,866). Bei der Dimension „Information“ des Zufriedenheitsfragebogens haben sich die Patienten in der IG nach 6 (*p* = 0,013) und 12 Monaten (*p* = 0,003) signifikant besser informiert gefühlt.

**Diskussion:**

Ein positiver Effekt der teambasierten Versorgung auf den Verlauf von Depression und Angst bei Patienten mit einer seropositiven RA im Krankheitsschub konnte gezeigt werden.

## Abstract (en)

**Background:**

At least 1 comorbidity occurs in 80% of patients with rheumatoid arthritis (RA). In addition to cardiovascular comorbidities psychological comorbid conditions are common. The prevalence of depression and anxiety is higher in patients than in the general population. Screening for comorbidities is crucial. A shortage of outpatient specialist care barely allows resources for this. The implementation of team-based care holds the potential to improve the standard of care while simultaneously working against the shortage of care.

**Objective:**

The aim of the study was to examine the effects of care on the course of depression and anxiety in patients with seropositive RA and active disease.

**Material and methods:**

A multicenter pragmatic randomized controlled trial was conducted over the course of 1 year with 224 patients. After baseline, five more visits followed. In the intervention group (IG), three were initially carried out by qualified rheumatological assistants. Depression, anxiety and patient satisfaction with outpatient care were looked at in detail.

**Results:**

In the IG the anxiety symptoms significantly improved over 12 months (*p* = 0.036). The proportions of patients with anxiety also significantly changed in the IG (*p* < 0.001), while there was no change in the control group between baseline and month 12. The values of the depression scale did not differ significantly (*p* = 0.866). In terms of the information dimension of the satisfaction questionnaire, patients in the IG felt significantly better informed after 6 months (*p* = 0.013) and 12 months (*p* = 0.003).

**Conclusion:**

A positive effect of team-based care on the course of depression and anxiety in patients with seropositive RA and active disease could be shown.

Durch chronisch entzündliche Erkrankungen steigt das Risiko psychischer Erkrankungen bei den Betroffenen. Zusätzlich zu den körperlichen Einschränkungen leiden viele Patienten unter Depressionen und/oder Angststörungen [1, 6]. Durch die bestehende Unterversorgung ist es im Versorgungsalltag häufig nicht möglich, ein umfassendes Screening auf Komorbiditäten zu gewährleisten. Der sichere Einsatz einer teambasierten Versorgungsform mit Delegation ärztlicher Leistungen an geschulte rheumatologische Fachassistenz (RFA) konnte gezeigt werden [12, 16–18]. Daten zu den Auswirkungen auf den Verlauf von Depression und Angst sind bislang begrenzt.

## Hintergrund und Fragestellung

Das Vorliegen einer Komorbidität bei Patienten mit einer rheumatoiden Arthritis (RA) ist häufig. Daten der Kerndokumentation des Deutschen Rheuma-Forschungszentrums Berlin (DRFZ) zeigen, dass je nach Altersstufe bei mindestens 80 % der Patienten eine oder mehrere Komorbiditäten auftreten [26]. Neben einer kardiovaskulären oder pulmonalen Komorbidität spielen auch psychische Erkrankungen eine bedeutende Rolle. Die Prävalenz der Depression im Zusammenhang mit einer RA liegt je nach Studie bei 9,5–41,5 % und das Risiko des Auftretens ist in den ersten 5 Jahren am höchsten [1]. Im Rahmen einer Metaanalyse aus 72 Studien konnte eine Prävalenz von 16,1 % ermittelt werden, wobei diese ebenfalls stark variierte und u. a. von dem jeweiligen Messinstrument abhing [19]. Auch bei der Ermittlung der Prävalenz in einem Früharthritiskollektiv konnte gezeigt werden, dass eine Depression mit im Vergleich zu der Normalbevölkerung signifikant höherer Prävalenz auftritt (16,5 % vs. 9,4 %) [8]. Das Auftreten einer Depression ist mit einer erhöhten Krankheitsaktivität assoziiert, korrespondierend nimmt auch die Schwere des subjektiven Krankheitsempfindens zu, wie z. B. die globale Patienteneinschätzung oder die Anzahl druckschmerzhafter Gelenke. Darüber hinaus kommt es zu niedrigeren Remissionsraten, einem schlechteren Therapieansprechen sowie höheren Krankheitskosten [1]. Nicht nur die Kosten der Erkrankung an sich steigen, sondern auch das Risiko von beruflichen Teilhabeeinschränkungen ist erhöht. Kosten im Zusammenhang mit Arbeitsunfähigkeiten sowie eingeschränkte berufliche Teilhabe, wie z. B. eine Erwerbsminderung, machen mit 39–86 % einen wesentlichen Anteil der gesamten Krankheitskosten aus [14]. Das Auftreten einer Depression stellt dabei den besten Prädiktor für eine Arbeitsunfähigkeit dar [4].

Während die Depression schon seit Längerem als Komorbidität bekannt ist, wurde eine Angststörung erst später als zusätzliches Problem erkannt. Wie bei der Depression zeigt sich mit einem berichteten Auftreten von 21–70 % eine hohe Spannbreite in den Angaben der Literatur [5]. Hier scheint insbesondere die Progredienzangst, also die Angst vor einem erneuten Schub oder Fortschreiten der Erkrankung, von hoher Relevanz für die Patienten zu sein. Im Vordergrund stehen die Angst vor Nebenwirkungen bei einer medikamentösen Langzeitbehandlung, Arbeitsunfähigkeit sowie Verlust der Autonomie [6]. Freier et al. 2019 berichten aus der Früharthritiskohorte eine 3fach erhöhte Angstsymptomatik als in der Normalbevölkerung bereits bei einem Erstbesuch (23,3 % vs. 6,8 %) [8]. Ein ausführliches und regelmäßiges Screening auf Komorbidität ist also hoch relevant: 1. aufgrund des Einflusses auf die rheumatische Grunderkrankung und 2. weil sie häufig ihrerseits behandlungsbedürftig ist. Dies wurde 2016 auch von der europäischen Fachgesellschaft European Alliance of Associations for Rheumatology (EULAR) aufgegriffen [2]. Allerdings besteht in der Rheumatologie nach wie vor eine Unterversorgung, die durch Mangel an Rheumatologen, einer regionalen Ungleichheit und einer Fehlallokation ärztlicher Ressourcen entsteht [28]. Häufig ist in der ärztlichen Sprechstunde keine Zeit mehr für das Screening auf Komorbidität vorhanden. Mittlerweile konnten die Vorteile einer teambasierten Versorgungsform mit Delegation ärztlicher Leistungen an geschulte rheumatologische Fachassistenz (RFA) in mehreren Studien auch in Deutschland aufgezeigt werden [12, 16–18]. Die rechtlichen Rahmenbedingungen sind abgesteckt, und seit 2021 liegt das Musterfortbildungscurriculum der Bundesärztekammer zur Aufstiegsqualifikation „Medizinische Fachangestellte für Rheumatologie vor“ (s. Überblick [13]). Die Implementierung einer strukturierten Visite durch die RFA bietet das Potenzial, die Versorgung von Patienten mit chronisch entzündlichen Erkrankungen zu verbessern und gleichzeitig der Unterversorgung zu begegnen.

Ziel der vorliegenden Arbeit war es, die Auswirkungen einer teambasierten Versorgungsform auf den Verlauf einer Depression und Ängstlichkeit bei Patienten mit einer seropositiven rheumatoiden Arthritis im Krankheitsschub zu untersuchen.

## Studiendesign und Untersuchungsmethoden

Die Daten wurden im Rahmen der randomisierten, kontrollierten, pragmatischen multizentrischen Studie: „Effektivität der RFA-Sprechstunde (ERFASS)“ zum Vergleich einer teambasierten Versorgung mit der Regelversorgung erhoben [12]. Eingeschlossen wurden volljährige Patienten mit einer vom Arzt gesicherten Diagnose einer Rheumafaktor- und/oder ACPA-positiven rheumatoiden Arthritis (ICD-10 M05.8) im Krankheitsschub zu entweder Therapiebeginn, Therapieumstellung oder bei Therapieeskalation. Ausgeschlossen wurden Patienten, die absehbar für eine 1‑jährige Nachbeobachtungsdauer nicht zur Verfügung standen, mit schweren Begleiterkrankungen nach Beurteilung des behandelnden Arztes, bei mangelnden Deutschkenntnissen und fehlender Einwilligungsfähigkeit. In dem 12-monatigen Beobachtungszeitraum fanden nach der Baselinevisite 5 Folgevisiten statt (Wochen 6, 12, 24, 36 und 52). Die Visiten in Woche 6, 12 und 36 wurden in der Interventionsgruppe zunächst durch die RFA durchgeführt, mit anschließendem kurzen Arztkontakt. Als primärer Endpunkt wurde die Veränderung des „Disease Activity Score 28“ (DAS28) über 12 Monate auf Nicht-Unterlegenheit untersucht [12].

Die sekundären Endpunkte beinhalten unter anderem das Vorliegen einer depressiven und/oder ängstlichen Symptomatik, gemessen mit der „Hospital Anxiety and Depression Scale“ (HADS) [10], sowie die Patientenzufriedenheit gemessen mit dem „Zufriedenheit in der ambulanten Versorgung – Qualität aus Patientenperspektive“(ZAP)-Fragebogen [3]. Der ZAP-Fragebogen ist ein standardisiertes Instrument zur Messung der prozessbezogenen Patientenzufriedenheit und besteht insgesamt aus 23 Items, die 4 Dimensionen zugeordnet sind: 8 Items Arzt-Patienten-Interaktion, u. a. Verständnis oder Einfühlungsvermögen, 8 Items Information (z. B. zu Ursachen oder Verlauf der Erkrankung), 4 Items Praxisorganisation (z. B. Wartezeit) sowie fachliche Kompetenz mit 3 Items (u. a. Gründlichkeit und Sorgfalt). Darüber hinaus gibt es 3 Globalfragen, welche die „Zufriedenheit mit dem Arzt insgesamt“, das Vertrauen zum Arzt sowie eine Einschätzung der Behandlungsqualität erfragen, die jeweils auf einer Likert-Skala von 1 bis 4 beantwortet werden. In der Studie wurde die originale Formulierung „Arzt/Ärztin“ durch die Begriffe „Behandler/Behandlerin“ ersetzt. Dazu wurden die Autoren des Fragebogens vorab kontaktiert.

Der HADS besteht aus 2 Subskalen mit je 7 Items, die jeweils auf einer Likert-Skala von 1 bis 4 beantwortet werden. Bei dem HADS handelt es sich um einen Fragebogen zur Selbstbeurteilung der Ausprägung ängstlicher und depressiver Symptomatik. Der Cut-off für sowohl die Depressions- als auch die Ängstlichkeitsskala des HADS lag bei ≥ 8 (milde/moderate Symptomatik) und der Schwellenwert für Sicherheitswarnungen bei ≥ 11 (abnormale Symptomatik). Die Fragebögen zu den sekundären Endpunkten wurden zu Baseline, Monat 6 und Monat 12 erhoben.

### Inhalte der Visiten

#### Interventionsgruppe

Für die RFA-Visite wurde ein Zeitfenster von 30 min eingeplant. Die Aufgaben umfassten die vorbereitende Anamnese gemäß einer Checkliste, Bestimmung der Krankheitsaktivität (DAS28), Screening auf Komorbidität sowie Informationen zu Medikamenteneinnahme und unerwünschte Medikamentenwirkungen. Ein weiterer Schwerpunkt der Visite lag in der Erfassung der Lebensumstände, psychischer Belastungen, Befragung zur Arbeitsfähigkeit und Ermittlung eines Bedarfs einer Rehabilitation oder anderer unterstützender Maßnahmen. Im Anschluss an das Gespräch mit dem Rheumatologen fand bei Bedarf eine Schulung zum Verständnis und zur Applikation der neuen Therapie statt.

#### Kontrollgruppe

Die Patienten in der Kontrollgruppe erhielten weiterhin die Regelversorgung mit Terminen bei dem behandelnden Rheumatologen alle 3 Monate für die je etwa 15–20 min eingeplant wurden. Zusätzlich fand ein kurzer Treat-to-Target(T2T)-Besuch statt.

In beiden Gruppen konnten die Patienten bei Problemen zusätzliche Termine bekommen.

### Fallzahlkalkulation

Basierend auf dem Wilcoxon-Vorzeichen-Rang-Test, wurde die Fallzahlkalkulation für die sekundären Endpunkte durchgeführt. Die Fallzahlkalkulation mit G*Power 3 hat bei einer Drop-out-Rate von 10 %, einem α von 0,025, einer Power von 95 sowie einer Effektgröße von d = 0,4 ergeben, dass mindestens 74 Patienten berücksichtigt werden müssen.

### Statistische Analysen

Statistische Analysen wurden mit der Software IBM SPSS Statistics V.25 (IBM, New York) durchgeführt.

Der Shapiro-Wilk-Test wurde verwendet, um auf Normalverteilung zu prüfen. Der t‑Test für abhängige Stichproben bzw. der Wilcoxon-Vorzeichen-Rang-Test und der t‑Test für unabhängige Stichproben bzw. der Mann-Whitney-U-Test wurden angewandt, um die Veränderung über die Zeit sowie die Nicht-Unterlegenheit bei einem Grenzwert von 0,4 zu untersuchen. Diese Effektgröße wird als minimaler klinisch relevanter Unterschied bei typischen „patient reported outcomes“ angesehen [23]. Um anteilige Veränderungen zu betrachten, kam der McNemar-Test zur Anwendung.

## Ethische und administrative Aspekte

Bei der ERFASS-Studie handelte es sich um ein Subprojekt des vom Innovationsfonds geförderten Projektes Rheuma-VOR (#01NVF16029). Die Studie wurde mit Zustimmung der zuständigen Ethikkommission der Medizinischen Hochschule Hannover durchgeführt (# 3638-2017), im Einklang mit nationalem Recht sowie gemäß der Deklaration von Helsinki von 1975 (in der aktuellen, überarbeiteten Fassung) durchgeführt. Die Rheuma-Liga Niedersachsen e. V. war in alle Schritte der Studie in Bezug auf die Planung, Auswertung und Diskussion der Ergebnisse involviert. Von allen beteiligten Patienten liegt eine Einverständniserklärung vor. Die Studie wurde beim deutschen Register für klinische Studien registriert (DRKS00013055).

## Ergebnisse

Es wurden 224 Patienten auf die Kontroll- und Interventionsgruppe randomisiert (113, 111). Die Drop-out-Rate betrug 8 % (s. Überblick [12]). Von 101 (KG) und 97 (IG) Patienten konnte der über alle Zeitpunkte vollständige Datensatz der Hospital Anxiety and Depression Scale ausgewertet werden. Die deskriptiven Ergebnisse zu Baseline sind in Tab. 1 aufgeführt.

**Table 1:** 

	KG (*n* = 113)	IG (*n* = 111)	Gesamt (224)
Weiblich. *n* (%)	86 (77)	80 (72)	166 (74)
Alter (Jahre), MW (SD)	58 (12)	59 (12)	59 (12)
RF-positiv (*n*)	105	101	206
ACPA-positiv (*n*)	96	96	192
Berufstätig, *n* (%) (112, 110)^a^	51 (46)	54 (49)	105 (47)
Schulbildung, *n* (%), (112, 110)^a^			
Keine weiterführende Schule	94 (83 %)	83 (75 %)	177 (79 %)
Weiterführende Schule	18 (17 %)	27 (25 %)	45 (21 %)
Ausbildung (111, 109), *n* (%)			
Keine	18 (16)	16 (15)	34 (16)
Berufsausbildung	85 (77)	79 (72)	164 (74)
Universitätsabschluss	8 (7)	14 (13)	22 (10)
Therapieregime *n* (%)			
Therapieeinstellung	41 (37)	28 (25)	69 (31)
Therapieumstellung	40 (36)	49 (44)	89 (40)
Therapieeskalation	31 (27)	34 (31)	65 (29)
Krankheitsdauer, Jahre, Median	6 (3–13)	8 (3–19)	6 (3–26)
Baselinetherapie, *n* (%) (103, 104)^a^			
Glukokortikoide	39 (38)	39 (38)	78 (38)
Methotrexat (103, 105)^a^	35 (34)	41 (39)	76 (37)
Leflunomide	14 (14)	11 (11)	25 (12)
Sulfasalazin	4 (4)	4 (4)	8 (4)
Hydroxychloroquin	4 (4)	2 (2)	6 (3)
JAK-Inhibitoren	2 (2)	0 (0)	2 (1)
Biologika	22 (21)	27 (26)	49 (22)
Ergebnisse, median (IQR)			
DAS28-CRP (110, 111)^a^	4,4 (3,5–5,1)	4,5 (3,4–5,2)	4,4 (3,5–5,2)
Druckschmerzhafte Gelenke	6 (2–10)	6 (2–12)	6 (2–11)
Geschwollene Gelenke	3 (1–6)	3 (1–6)	3 (1–6)
Patienteneinschätzung Krankheitszustand	60 (44–75)	60 (40–79)	60 (42–75)
HADS Median (112, 109)			
Depression (Median)	6 (3–9)	4 (2–8)	5 (2–8)
Ängstlichkeit (Median)	6 (3–9)	6 (3–10)	6 (3–9)
HADS Anteile (112, 109) *n* (%)			
Ängstliche Symptome			
Keine	71(63 %)	69 (63 %)	140 (63 %)
Mild/moderat	24 (22 %)	19 (18 %)	43 (20 %)
Abnormal	17 (15 %)	21 (19 %)	38 (17 %)
Depressive Symptomatik			
Keine	69 (62 %)	81 (74 %)	150 (68 %)
Mild/moderat	26 (23 %)	13 (12 %)	39 (18 %)
Abnormal	17 (15 %)	15 (14 %)	32 (14 %)
ZAP Globalfragen (Median)			
Vertrauen (112, 110)^a^	4 (3–4)	4 (4–4)	4 (3–4)
Qualität (110, 109)^a^	2 (2–3)	3 (2–3)	3 (2–3)
Zufriedenheit (110, 109)^a^	2 (2–3)	3 (2–3)	3 (2–3)
Dimension Information (111, 108)^a^ (MW)	85 (SD 16)	87 (SD 15)	86 (SD 15)

*ACPA* Antikörper gegen citrullinierte Proteine, *DAS28-CRP* Disease Activity Score in 28 Gelenken gemessen mit CRP, *HADS* Hospital Anxiety and Depression Scale, *IG* Interventionsgruppe, *KG* Kontrollgruppe, *MW* Mittelwert, *RF* Rheumafaktor, *SD* Standardabweichung, *ZAP* Fragebogen zur Zufriedenheit in der ambulanten Versorgung

^a^Die Zahlen der zur Verfügung stehenden Daten sind die der randomisierten Gruppen, 113 in der KG und 111 in der IG, wenn in Klammern nichts anderes angegeben wurde

**Figure 1:**
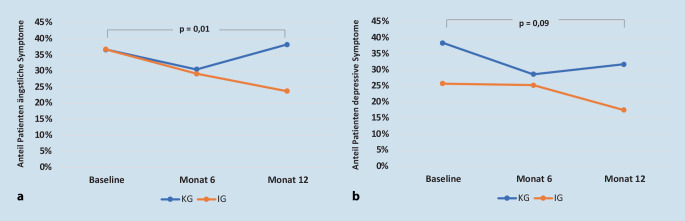

In Bezug auf die Veränderung der Ängstlichkeit der Patienten konnte eine signifikante Verbesserung des Scores in der Interventionsgruppe nachgewiesen werden, in der Kontrollgruppe jedoch nicht. Zu Baseline gaben in der Interventions- sowie Kontrollgruppe 37 % der Patienten moderate bis ausgeprägte ängstliche Symptome an (entsprechend eines HADS Scores ≥ 8). Zu Monat 6 sank dieser Anteil in beiden Gruppen, und zwar auf 29 % (IG) bzw. 30 % (KG). In der IG ging der Anteil nach 12 Monaten weiter zurück auf 24 % (*p* < 0,001), in der KG stieg er wieder auf 38 % an (*p* = 0,5) Der Unterschied zwischen den Gruppen ist signifikant (*p* = 0,035, Tab. 2; Abb. 1a).

**Table 2:** 

	KG	IG	Differenz		
	Median (IQR)^a^	Median (IQR)^a^	U	z	*p*-Wert*
*HADS‑D*					
Baseline (112, 109)^b^	6 (3, 9)	4 (2, 8)	–	–	–
Monat 12 (101, 97)^b^	5 (2, 9,50)	3 (1, 6)	4781,000	−0,169	0,866
*HADS‑A*					
Baseline (112, 109)^b^	6 (3, 9)	6 (3, 10)	–	–	–
Monat 12 (102, 97)^b^	6 (2, 9)	4 (2, 7)	4056,000	−2,101	0,036
*ZAP Globalfragen*					
Vertrauen zu Behandler/Behandlerin					
Baseline (112, 110)^b^	4 (3, 4)	4 (4, 4)	–	–	–
Monat 12 (102, 97)^b^	4 (3, 4)	4 (4, 4)	4856,500	−0,285	0,775
Behandlungsqualität					
Baseline (110, 109)^b^	2 (2, 3)	3 (2, 3)	–	–	–
Monat 12 (102, 97)^b^	3 (2, 3)	3 (3, 3)	4506,500	−1,074	0,283
Zufriedenheit insgesamt					
Baseline (110, 109)^b^	2 (2, 3)	3 (2, 3)	–	–	–
Monat 12 (102, 97)^b^	3 (2, 3)	3 (3, 3)	4722,000	−0,399	0,690
*Dimension Information*					
Baseline	85 (SD 16)	87 (SD 15)	–	–	–
Monat 6	85 (SD 16)	90 (SD 14)	–	–	–
Monat 12	84 (SD 17)	91 (SD 12)	–	–	0,003

*z* z-Score, *U* Mann-Whitney (U)-Statistik, *HADS‑D* Hospital Anxiety and Depression Scale, Ausprägung Depression, *HADS‑A* Hospital Anxiety and Depression Scale, Ausprägung Angst, *IG* Interventionsgruppe, *KG* Kontrollgruppe, *SD* Standardabweichung, *ZAP* Fragebogen zur Zufriedenheit in der ambulanten Versorgung

*p-Werte gemäß Nicht-Unterlegenheit der Veränderung der Scores (bei einem Grenzwert von 0,4)

^a^Median der beobachteten Werte (nicht Änderung)

^b^Die Zahlen der zur Verfügung stehenden Daten sind die der randomisierten Gruppen, 113 in der KG und 111 in der IG, wenn in Klammern nichts anderes angegeben wurde

Der Verlauf der von den Patienten berichteten depressiven Symptomatik hat sich in beiden Gruppen signifikant verbessert (*p* = 0,001), zwischen der Interventions- und Kontrollgruppe allerdings nicht unterschieden (*p* = 0,866, Tab. 2). In der Kontrollgruppe kam es nach einem initialen Abfall von 38 auf 29 % des Anteils der Patienten mit einer depressiven Symptomatik nach Monat 6 wieder zu einem leichten Anstieg (32 %). In der Interventionsgruppe blieb dahingegen der Anteil zunächst in etwa gleich (26 %, 25 %), nach Monat 6 kam es jedoch zu einem deutlichen Abfall des Anteils der Patienten mit einer depressiven Symptomatik (18 %). Diese Veränderungen sind weder in der gesamten Studienpopulation (*p* = 0,092) noch in der Kontrollgruppe (*p* = 0,324) oder der Interventionsgruppe (*p* = 0,090) signifikant (Abb. 1b).

**Figure 2:**
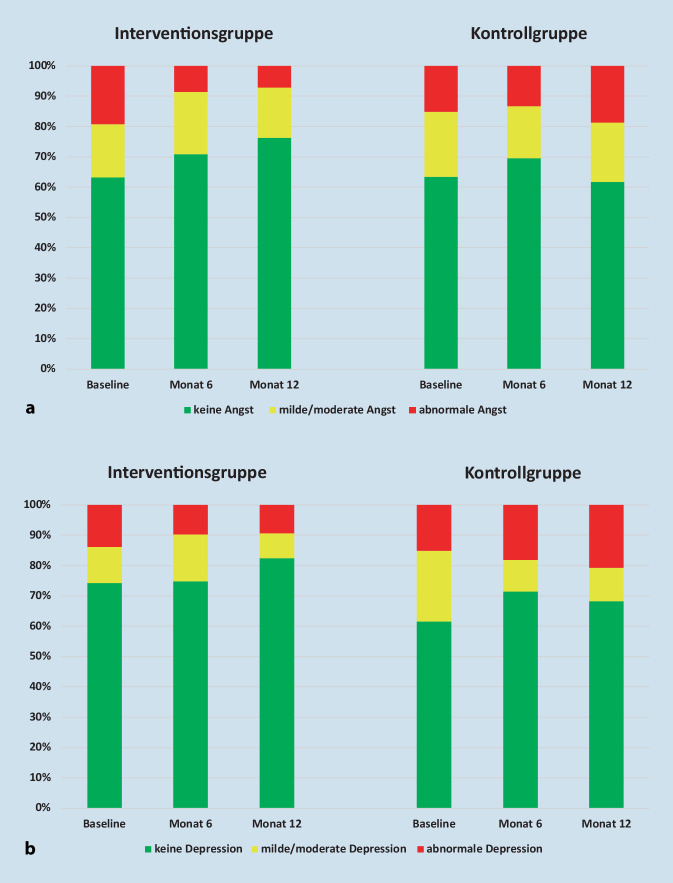

Die Anteile der Patienten mit einer milden/moderaten Angstsymptomatik bleiben in beiden Gruppen über den Zeitverlauf nahezu unverändert, die Veränderung findet vornehmlich in der Gruppe der Patienten statt, die eine abnormale Angstsymptomatik berichten (Abb. 2a). Die depressive Symptomatik verändert sich dahingegen in allen 3 Ausprägungen (Abb. 2b).

Hinsichtlich der Veränderung der 3 Globalfragen des ZAP über 12 Monate bezüglich des Vertrauens zu den Behandelnden (*p* = 0,775), der Qualität der Behandlung im Allgemeinen (*p* = 0,283) sowie der Zufriedenheit mit dem zuletzt besuchten Behandelnden im Allgemeinen (*p* = 0,690) wurden keine signifikanten Unterschiede zwischen den Gruppen festgestellt (Tab. 2). Bei der Zufriedenheit mit den Behandelnden in Bezug auf die Dimension der Information haben sich die Patienten in der IG signifikant besser informiert gefühlt als in der KG (*p* = 0,03, Tab. 2).

## Diskussion

Im Rahmen der ERFASS-Studie konnte insgesamt ein positiver Effekt einer teambasierten Versorgungsform auf den Verlauf einer patientenberichteten Symptomatik einer Depression und Ängstlichkeit bei Patienten mit einer Rheumafaktor- und/oder ACPA-positiven rheumatoiden Arthritis im Krankheitsschub nachgewiesen werden.

Informationen und Wissen können helfen, Angst zu reduzieren, und Selbstregulierung ist dabei ein wichtiger Aspekt im Umgang mit chronischen Erkrankungen [27]. Diese Selbstregulierung kann beigebracht und gelernt werden [25], und hier liegt ein wichtiger Ansatzpunkt für eine RFA-Visite. Durch die Visite bei der RFA können die Patienten viel über ihre Erkrankung und den Umgang damit lernen. Hilfestellung bei der Applikation der medikamentösen Therapie ist ein wichtiger Punkt, aber auch Informationen dazu, wie eine richtige Ernährung und Bewegung dabei helfen können, die Krankheit zu bewältigen. Dieses erhöhte Wissen und das Erlernen von Fähigkeiten können ihnen eine gewisse Sicherheit geben und dadurch positive Auswirkungen auf die Bewältigung der Angst haben [9]. Dies könnte erklären, warum lediglich die Angst und nicht die Depression signifikant besser geworden ist. Darüber hinaus konnte anhand inflammatorischer Wirkmechanismen gezeigt werden, dass ein signifikanter Zusammenhang zwischen RA und Depressionen besteht [5, 7, 22]. Bei Angststörungen konnten bisher keine direkten Zusammenhänge nachgewiesen werden, jedoch indirekte Zusammenhänge, die zeigen konnten, dass der Einfluss von psychologischem Stress auf Angststörungen durch inflammatorische Zytokine mediiert werden kann [15]. Der psychologische Stress kann durch die verbesserte Selbstwirksamkeit der Patienten gelindert werden, worüber im Verlauf auch die Angststörung reduziert werden kann. Die Behandlung der immunologischen Auslöser der Erkrankung kann auch einen positiven Einfluss auf die Symptome der Depression haben [22]. Da sich die Krankheitsaktivität in beiden Gruppen jedoch nicht signifikant über die Zeit unterscheidet, ist es auch naheliegend, dass sich die depressive Symptomatik ähnlich verändert.

**Figure 3:**
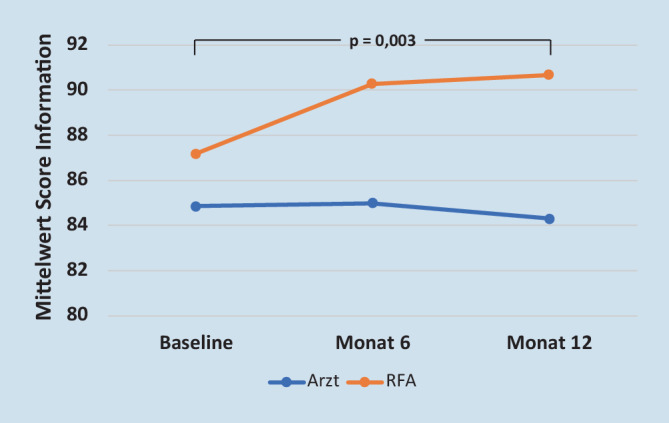

In einer von Meisters et al. [20] veröffentlichten Studie wurden Versorgungslücken aus Patienten- und Rheumatologensicht nach EULAR-Kriterien in 35 europäischen Ländern untersucht. Wichtige von den Patienten erwähnte Versorgungslücken sind unter anderem: Erhalt von ausreichenden Informationen bezüglich der Erkrankung und verschiedener Behandlungsmöglichkeiten sowie ein Eingehen auf die Patientenbedürfnissen. Im Rahmen der ERFASS-Studie konnte gezeigt werden, dass sich die Patienten in der teambasierten Versorgungsform signifikant besser informiert gefühlt haben (Abb. 3). Im Gegensatz zu der RFA-Visite bietet die reguläre Sprechstunde für eine ausgedehnte Schulung der Patienten keine Zeit, wodurch erklärt werden könnte, warum sich in der Kontrollgruppe die Angst nicht signifikant verbessert. Als zusätzliche Unterstützung und Vertiefung der Interaktion könnte der Einsatz von Digitalen Gesundheitsanwendungen (DiGAs) bei Angst und Depression überlegt werden. Patienten haben so die Möglichkeit, nach Einführung durch die RFA eigenständig zu Hause die Anwendung durchzuführen und bei Bedarf regelmäßig zu besprechen.

Auffällig ist, dass die Veränderung zwischen den beiden Gruppen zwischen Monat 6 und Monat 12 geschieht. Eine Erklärung hierfür könnte sein, dass die Patienten bislang in den meisten Fällen noch keine Erfahrung in der zusätzlichen Versorgung durch eine RFA hatten und dem zunächst skeptisch gegenüberstanden. Der Mehrwert der teambasierten Versorgung wurde dann erst im Verlauf erkannt.

Unsere Ergebnisse entsprechen internationalen Studien, die ebenfalls zeigen konnten, dass Visiten bei RFAs zu signifikanten Verbesserungen des psychischen Wohlergehens führen können [11, 21, 24].

Die Studie ist mit einigen Limitationen verbunden. Es wurden nur Patienten mit einer seropositiven RA im Krankheitsschub in die Studie eingeschlossen. Eine Übertragbarkeit auf andere Indikationen ist nicht uneingeschränkt möglich. Eine weitere Limitation ist, dass die Randomisierung der Patienten innerhalb der jeweiligen Zentren stattfand, und nicht als einzelne Cluster. Somit kann ein Halo-Effekt nicht grundsätzlich ausgeschlossen werden. Darüber hinaus fand entsprechend den rechtlichen Rahmenbedingungen immer ein Arztkontakt statt. Gemäß Studienprotokoll orientierte sich die Länge des Kontaktes u. a. an den Bedürfnissen des Patienten. In der teambasierten Versorgung steht der Arzt selbstverständlich ebenfalls als Ansprechpartner den Patienten zur Verfügung. Es konnte jedoch eine deutliche Zeitersparnis aufseiten der Ärzte nachgewiesen werden [12], sodass umfassende Information aufseiten der Rheumatologen eher unwahrscheinlich ist.

## Fazit für die Praxis

Die Einbeziehung einer 30-minütigen RFA-Visite bei ambulanten Wiedervorstellungen von Patienten mit einer RA hat zu einer Reduktion ängstlicher Symptome und zu einem Informationsgewinn bei den Betroffenen geführt. Neben der Zeitersparnis für die ärztlichen Tätigkeiten unterstreicht diese Verbesserung in der Ergebnisqualität die Versorgungsrelevanz der Delegation ärztlicher Leistungen in der Rheumatologie durch RFA.

## References

[1] Baerwald C, Manger B, Hueber A (2018). Depression als Komorbidität bei rheumatoider Arthritis. Z Rheumatol.

[2] Baillet A, Gossec L, Carmona L (2016). Points to consider for reporting, screening for and preventing selected comorbidities in chronic inflammatory rheumatic diseases in daily practice: a EULAR initiative. Ann Rheum Dis.

[3] Bitzer EM, Dierks ML, Dörning H (1999). Zufriedenheit in der Arztpraxis aus Patientenperspektive – Psychometrische Prüfung eines standardisierten Erhebungsinstrumentes. Z. Gesundheitswiss..

[4] Callhoff J, Albrecht K, Schett G (2015). Depression is a stronger predictor of the risk to consider work disability in early arthritis than disease activity or response to therapy. RMD Open – Rheum Muscoloskeletal Dis.

[5] Covic T, Cumming SR, Pallant JF (2012). Depression and anxiety in patients with rheumatoid arthritis: prevalence rates based on a comparison of the Depression, Anxiety and Stress Scale (DASS) and the hospital, Anxiety and Depression Scale (HADS). BMC Psychiatry.

[6] Engst-Hastreiter U, Duran G, Henrich G (2004). Progredienzangst (PA) bei chronischen Erkrankungen (rheumatischen Erkrankungen, Krebserkrankungen und Diabetes mellitus). Entwicklung eines psychologischen Fragebogens und eines Gruppenpsychotherapie-Programms. Akt Rheumatol.

[7] Felger JC, Lotrich FE (2013). Inflammatory cytokines in depression: neurobiological mechanisms and therapeutic implications. Neuroscience.

[8] Freier D, Englbrecht M, Höhne-Zimmer V (2019). Höhere Prävalenz von depressiven und ängstlichen Symptomen bei Früharthritispatienten im Vergleich zur Normalbevölkerung (Higher prevalence of depressive and anxiety symptoms in early arthritis patients in comparison to the normal population). Z Rheumatol.

[9] Goldberg RJ (1982). Anxiety reduction by self-regulation: theory, practice, and evaluation. Ann Intern Med.

[10] Herrmann C, Buss U, Lingen R (1994). The screening for anxiety and depression in routine medical care. Dtsch Med Wochenschr.

[11] Hill J, Thorpe R, Bird H (2003). Outcomes for patients with RA: a rheumatology nurse practitioner clinic compared to standard outpatient care. Musculoskelet Care.

[12] Hoeper JR, Zeidler J, Meyer SE (2021). Effect of nurse-led care on outcomes in patients with APCA/RF-positive rheumatoid arthritis with active disease undergoing treat-to-target: a multicenter randomized controlled trial. RMD Open – Rheum Muscoloskeletal Dis.

[13] Hoeper JR, Schuch F, Hoeper K (2023). Delegation in der Rheumatologie: Aktueller Stand und Perspektiven. Arthritis Rheuma.

[14] Hsieh P-H, Wu O, Geue C (2020). Economic burden of rheumatoid arthritis: a systematic review of literature in biologic era. Ann Rheum Dis.

[15] Khandaker GM, Zammit S, Lewis G (2016). Association between serum C-reactive protein and DSM-IV generalized anxiety disorder in adolescence: findings from the ALSPAC cohort. Neurobiol Stress.

[16] Kiltz U, Spiller I, Sieper J (2020). Is it possible to delegate medical services to qualified nurses specialized in rheumatology when evaluating patients with suspicion of ankylosing spondylitis?-Results of the PredAS study. Z Rheumatol.

[17] Krause D, Mai A, Denz R (2022). The structured delegation of medical care services for patients with inflammatory rheumatic diseases. Dtsch Ärztebl Int.

[18] Krueger K, Eder R, Mueller C (2018). OP0137 Assessing the risk of ra patients for comorbid conditions through a structured nurse-led interview – the eriko study.

[19] Matcham F, Rayner L, Steer S (2013). The prevalence of depression in rheumatoid arthritis: a systematic review and meta-analysis. Baillieres Clin Rheumatol.

[20] Meisters R, Putrik P, Ramiro S (2020). EULAR/eumusc.net standards of care for rheumatoid arthritis: cross-sectional analyses of importance, level of implementation and care gaps experienced by patients and rheumatologists across 35 European countries. Ann Rheum Dis.

[21] Ndosi M, Lewis M, Hale C (2014). The outcome and cost-effectiveness of nurse-led care in people with rheumatoid arthritis: a multicentre randomised controlled trial. Ann Rheum Dis.

[22] Nerurkar L, Siebert S, McInnes IB (2019). Rheumatoid arthritis and depression: an inflammatory perspective. Lancet Psychiatry.

[23] Norman GR, Sloan JA, Wyrwich KW (2003). Interpretation of changes in health-related quality of life: the remarkable universality of half a standard deviation. Med Care.

[24] Primdahl J, Sorensen J, Horn H (2014). Shared care or nursing consultations as an alternative to rheumatologist follow-up for rheumatoid arthritis outpatients with low disease activity—patient outcomes from a 2-year, randomised controlled trial. Ann Rheum Dis.

[25] Shahabi L, Naliboff BD, Shapiro D (2016). Self-regulation evaluation of therapeutic yoga and walking for patients with irritable bowel syndrome: a pilot study. Psychol Health Med.

[26] Thiele K, Albrecht K, Kopplin N (2022). Standardpräsentation 2020. Daten aus der Kerndokumentation.

[27] Weidner G, Sieverding M, Chesney MA (2016). The role of self-regulation in health and illness. Psychol Health Med.

[28] Zink A, Braun J, Gromnica-Ihle E (2017). Memorandum der Deutschen Gesellschaft für Rheumatologie zur Versorgungsqualität in der Rheumatologie – Update 2016. Z Rheumatol.

